# Spontaneous Charging of Drops on Lubricant-Infused Surfaces

**DOI:** 10.1021/acs.langmuir.2c02085

**Published:** 2022-10-03

**Authors:** Shuai Li, Pravash Bista, Stefan A. L. Weber, Michael Kappl, Hans-Jürgen Butt

**Affiliations:** Max Planck Institute for Polymer Research, Ackermannweg 10, 55128Mainz, Germany

## Abstract

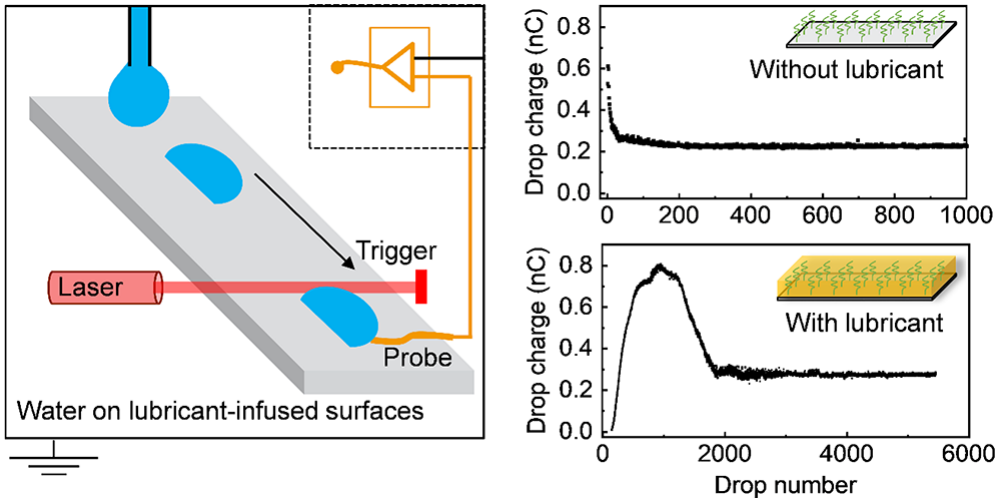

When a drop of a polar liquid slides over a hydrophobic surface, it acquires a charge. As a result, the surface charges oppositely. For applications such as the generation of electric energy, lubricant-infused surfaces (LIS) may be important because they show a low friction for drops. However, slide electrification on LIS has not been studied yet. Here, slide electrification on lubricant-infused surfaces was studied by measuring the charge generated by series of water drops sliding down inclined surfaces. As LIS, we used PDMS-coated glass with micrometer-thick silicone oil films on top. For PDMS-coated glass without lubricant, the charge for the first drop is highest. Then it decreases and saturates at a steady state charge per drop. With lubricant, the drop charge starts from 0, then it increases and reaches a maximum charge per drop. Afterward, it decreases again before reaching its steady-state value. This dependency is not a unique phenomenon for lubricant-infused PDMS; it also occurs on lubricant-infused micropillar surfaces. We attribute this dependency of charge on drop numbers to a change in surface conductivity and depletion of lubricant. These findings are helpful for understanding the charge process and optimizing solid–liquid nanogenerator devices in applications.

## Introduction

Slide electrification^1−6^ is the spontaneous charging of hydrophobic, insulating surface by sliding liquid drops. It is generally accepted that drops of polar liquids, such as water, moving down inclined, with low-energy and low-conductivity surfaces, acquire a charge. Usually they charge up positively, while the negative countercharge is deposited on the free solid surface. This may be attributed to ion transfer to the solid surface, e.g., OH^–^.^7−9^ Despite being ubiquitous and despite potential applications in the generation of electric energy or manipulating drop movement,^10−13^ we have little fundamental understanding of slide electrification. There is no clear picture of the underlying microscopic processes nor a first-principles predictive model. With respect to solid tribocharging,^14−16^ one fundamental difference is that, in slide electrification, no huge shear stresses can occur, not even locally. In solid tribocharging, protrusions on the microscale typically experience huge shear stresses. They can break covalent bonds and generate locally enough energy to bring electric charges to the free solid surface. Such a high shear stress cannot be generated by liquid drops.

To better understand the underlying principles of slide electrification, we study charge separation on lubricant-infused surfaces (LISs). In case of LIS, the wetted surface is liquid rather than solid. Usually, LIS consist of a structured surface that is impregnated with a lubricant.^17−22^ These surfaces have attracted much attention because they provide low sliding angles. Water drops start sliding down inclined LIS even at low tilt angles. However, LISs usually suffer from a depletion problem, which may affect the electrification efficiency.^23^ To better understand the slide electrification on LISs, we measured the drop charge of the sliding deionized water drops on lubricant-infused PDMS with different oil content, as well as the conventional LIS that use a structured surface.

With respect to slide electrification, LIS are interesting for three reasons. From the fundamental point of view, they reduce shear at the substrate–liquid interfaces. Second, they allow drops to slide at low tilt angles, which, for electric energy generation, may be important. Third, as it will turn out, charge measurements provide an easy method to detect the depletion of LIS.

## Experimental Section

### Preparation of Lubricant-infused Surfaces

#### Cleaning and Activation of Glass Substrates

Glass substrates (2 mm thick) were washed ultrasonically for 15 min in toluene, ethanol, and water, respectively, before blow-drying by nitrogen. The substrates then were treated with an oxygen-plasma (Diener Electronic Femto, 120 W, with an oxygen flow rate of 6 cm^3^ min^–1^) for 5 min.

#### Preparation of PDMS Surfaces

To prepare polydimethylsiloxane (PDMS) surfaces,^24^ the substrates were immersed in 40 mL toluene (with saturated water) mixed with 1.4 mL dimethyldichlorosilane. After reacting for 0.5 h, the substrates were rinsed with toluene to remove the residues and dried with nitrogen. The PDMS coating was ca. 4 nm with a surface roughness of ca. 1 nm, which were measured using atomic force microscopy (AFM).

#### Preparation of SU8 Surfaces

To prepare SU8 pillars,^25,26^ SU8 photoresist was spin-coated on the substrates first. Then the SU8 film was cured into a structured array pattern of micropillars (pillar diameter, 5 μm; center-to-center distance, 10 μm; height: 10 μm), utilizing photolithography. After UV exposure (8 s) using a photomask and baking cycles at 65 °C (30 min), 95 °C (3 min) and 65 °C (30 min), the uncured SU-8 was dissolved in a developer solution and washed in propanol. The SU8 was then coated with silica by treatment with an O_2_ plasma for 30 s, followed by immersion in a solution of tetraethoxysilane (1.82 mL) and ammonium hydroxide (28% in water, 4.2 mL) in ethanol (50 mL) for 2–3 h. After 1 h of oxygen plasma, the filament was coated on the SU8 pillar by immersing the substrate to a solution which contains 0.4 mL trichloromethylsilane and 100 mL toluene (with 150 ppm water). Finally, PFOTS was coated on the SU8 surfaces by placing the glass substrates into a desiccator, where 20 μL trichloro(1H,1H,2H,2H-perfluorooctyl)silane (PFOTS) was deposited inside. The chamber was then evacuated to a pressure of 50 mbar and the reaction was allowed to proceed for 3 h.

#### Preparation of Lubricant-Infused Surfaces

To prepare lubricant-infused surfaces,^27,28^ a 200 μL drop of silicone oil (Fisher Scientific UK, 100 cSt) were deposited on the substrate. Then, the substrates were put vertically in a glass staining tank with a naturally tilted angle of 90° ± 2°. The lubricant content/thickness was controlled by the tilt time. Afterward, the substrates were put horizontally in the sample box before further measurements.

### Surface Characterization

#### Thickness Measurement

The thickness of the lubricant on the PDMS was measured by confocal laser scanning microscopy (Leica SP8). Lumogen red (F300 BASF) was used to dye the lubricant (concentration: 0.1 mg/mL).^29^ The microscope was equipped with a C-Apochromat 40/1.2 W water-immersion objective to visualize the thickness of the lubricant. For excitation, an argon laser fiber-coupled to the microscope were used (633 nm). Each measurement was conducted on more than 10 positions.

#### Contact Angles

Advancing and receding contact angles were measured using an OCA 35 contact angle goniometer (Dataphysics, Germany) in the sessile drop configuration. The water volume was gradually (1 μL s^–1^) increased from 10 to 20 μL and then decreased from 10 to 20 μL, respectively. On surfaces without lubricant, the contour of the drop was easily detected by the software. On lubricant-infused surfaces, wetting ridge formed immediately after the water drop was deposited on the surface. To extract the apparent contact angles, the interface between water drop and the surface was defined to be the position which is slightly above the meniscus, to ignore the distortion effect of the wetting ridge.^30^

#### Charge Measurement

Slide electrification experiments were conducted in a custom-built device (Figure 2a, presented later in this work) using deionized water. The system mainly consists of a Faraday cage, a water pump, a current amplifier, and a LabVIEW program.^3^ The Faraday cage was electrically grounded, and inside there are a tilted stage, a flat-tipped syringe needle, a laser and its detector, and an ionizing air blower. The surfaces were mounted on the tilted stage at 50°, and the needle (drop volume: 45 μL) was mounted 5 mm above the surface. The deionized water drops (Sartorius Arium Pro VF, 18.2 MΩ cm resistivity, Germany) were generated regularly at a rate of 30 drops/min by the water pump (Gilson Minipuls 3, Middleton, WI, USA). The drops were deposited on the top area of the tilted surfaces. As drops slid down the surface, they contacted two electrodes and a laser beam. The first electrode grounded the drop to ensure that it starts electrically neutral. The second electrode measured the drop current using a low noise current amplifier (response time = 5 μs, FEMTO DLPCA-200, Berlin, Germany). By integrating the current signal over the peak (see Figure S1 in the Supporting Information, 0–2 ms), a drop charge was obtained. The laser beam was used to trigger the data collection and a National Instruments data acquisition card (NI USB-6366 X-Series) was used to record the discharge current by the LabVIEW program. Before every new experiment, an ionizing air stream (Simco-Ion, Hatfield, PA, USA) was blown over the surface for 5 min in order to neutralize the surface. Drops then run over the surfaces, and for every drop, a current spike was recorded when it touched the second electrode. Current signals were integrated for every drop to quantify the drop charge.

#### Velocity Measurement

To measure the drop velocity on the surfaces another custom built setup was used. A high-speed camera (FASTCAM MINI UX100, Photron with a Titan TL telecentric lens, 0.268×, 1″ C-Mount, Edmund Optics) with a frame rate of 500 FPS was used to record the sliding drops on the tilted surfaces. A MATLAB program (DSAfM) was adopted to analyze the video. To obtain the drop velocity images were further analyzing, first, they were corrected by subtracting the background from the original images. Afterward, the edge position of the drops were identified and finally the velocities were calculated from the edge positions in each frame (for details, see ref (6)).

## Results and Discussion

### Surface Characterization

**Figure 1:**
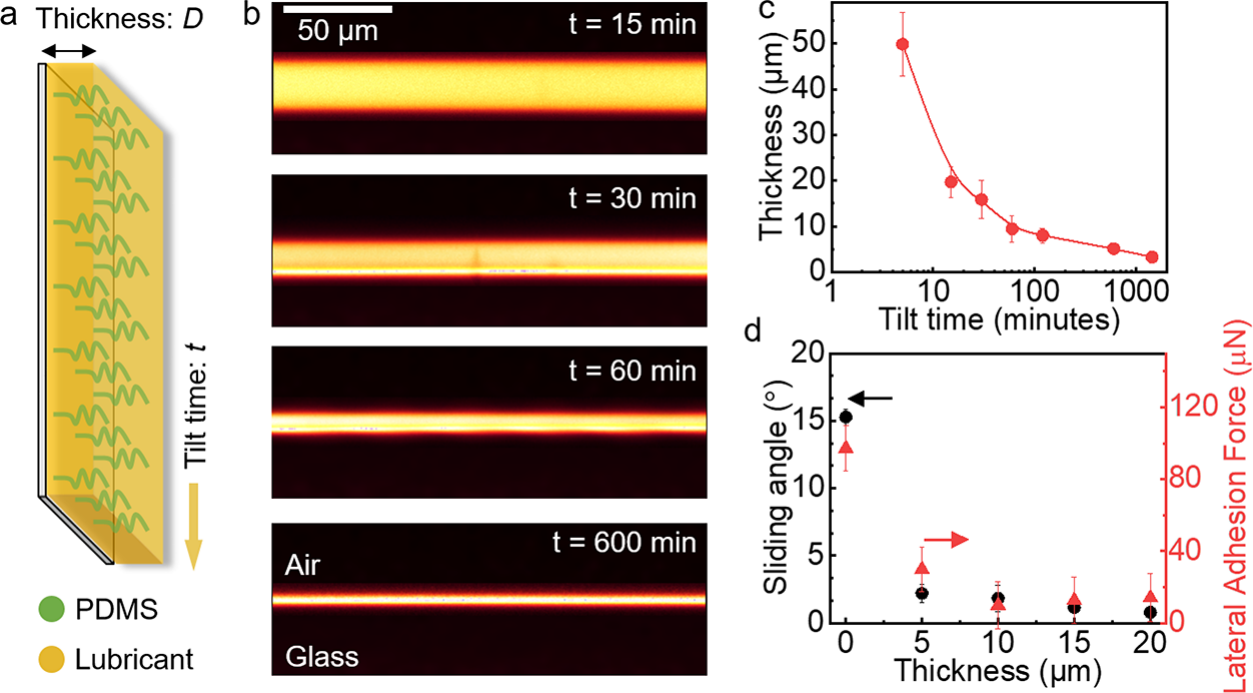
Surface characterization. (a) Schematic of lubricant-infused PDMS surfaces. After depositing a dyed lubricant on the PDMS surfaces, the samples were tilted vertically for different time *t* to remove the residual lubricant. After that, the samples were stored carefully before further measurements. (b) Confocal microscope images (vertical cross sections) of PDMS surfaces with lubricant after different tilt times *t*. The images represent vertical cuts and are used to measure the lubricant film thickness. (c) Lubricant thickness as a function of tilt time. (d) Sliding angle and calculated lateral adhesion force of a 45 μL water drop on PDMS surfaces with different lubricant thickness.

To control the thickness of the lubricant layer, the glass plates were placed vertically (Figure 1a, as well as Figure S2 in the Supporting Information) for 15, 30, 60, or 600 min, resulting in a lubricant thickness of ca. *D* = 20, 15, 10, 5 μm, respectively (Figures 1b and 1c). The lubricant thickness was obtained by plotting the intensity curve of the lubricant (Figure S3 in the Supporting Information). The results in Figure 1c show that the lubricant thickness was relatively homogeneous all over the surface within a small error. For convenient description here, PDMS-*x* are used to denote the PDMS surface with the lubricant of *x* μm. For example, PDMS-20 represents the PDMS surface with lubricant thickness of 20 μm.

Because of the flexible polymer chains, PDMS-0 showed a contact angle hysteresis with water of 15° ± 2° (Table 1) and a sliding angle of 15° ± 1° (Figure 1d). When adding lubricant, the contact angle hysteresis further decreased and water drops started to move at even lower tilt angles. For PDMS-20, the contact angle hysteresis and sliding angle for water was 2° ± 1° and 1° ± 1°, respectively. The low contact angle hysteresis on PDMS surfaces also lead to a low adhesion force for water drops. The lateral adhesion force can be calculated by^31−33^
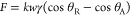
1where *k* ≈ 1, *w*, γ, θ_R_, θ_A_ are the dimensionless factor, droplet contact width, surface tension of the liquid, receding and advancing contact angle, respectively. As shown in Figure 1d, without lubricant, a 45 μL water drop showed a lateral adhesion force of 97 μN ± 12 μN. When adding lubricant, the lateral adhesion force decreased to below 40 μN. All the results above illustrate the high mobility of water drops on lubricant-infused PDMS surfaces.

**Table 1:** Contact Angle of Water on Lubricant-Infused Surfaces

surface	advancing contact angle, θ_A_ (deg)	receding contact angle, θ_R_ (deg)	contact angle hysteresis, Δθ (deg)
PDMS-0	108 ± 1	92 ± 1	15 ± 2
PDMS-5	103 ± 1	98 ± 2	5 ± 2
PDMS-10	98 ± 2	96 ± 2	2 ± 1
PDMS-15	98 ± 1	96 ± 1	2 ± 1
PDMS-20	96 ± 1	94 ± 2	2 ± 1
SU8	144 ±. 2	136 ± 3	8 ± 1
lubricant-infused SU8	97 ± 1	95 ± 1	3 ± 1

For comparison, we also studied a model lubricant-infused array of micropillars (Figure S4 in the Supporting Information for schematic and SEM images of the SU8 pillars). The contact angle hysteresis for water on SU8 with and without lubricant were 8° ± 1° and 3° ± 1°, respectively (Table 1).

### Slide Electrification

**Figure 2:**
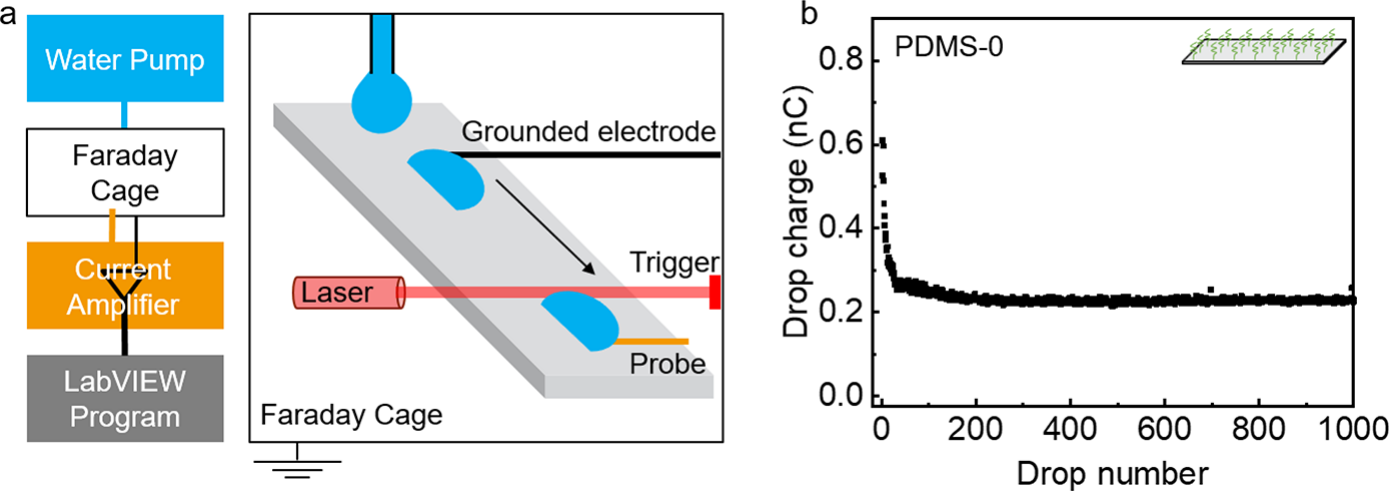
Slide electrification. (a) Schematic of the device for charge measurement. Its main components are a water pump, a Faraday cage, a current amplifier, and a LabVIEW program for analysis. The drop current is measured by an electrode, which is then amplified for analysis. (b) Drop charge versus drop number on PDMS-0. The drop charge is obtained by integrating the current over the first 2 ms.

Slide electrification experiments were conducted in a custom build device (Figure 2a) using deionized water. Figure 2b shows one representative charge curve on a PDMS surface (charge per drop versus drop number, *Q* vs *n*). The first drop carried the highest charge of ca. 0.61 nC. After the first drop, the charge of successive drops decreased. After ca. 40 drops, it reached a steady-state value of 0.23 nC ± 0.01 nC. This is consistent with previous study on hydrophobic surface.^3,34^ Because surface is neutral in the beginning, the first drop slides over the surface and has the highest charge value. After the first drop leave, the surface discharges within a characteristic time τ. Since the surface is not fully neutralized before the following drop slide on the surface, the next drop accumulates less charge. So the charge value per drop is influenced by two processes, charge neutralization process and accumulation process. When the two processes reach a dynamic equilibrium state, the charge is saturated at a steady state, e.g., in Figure 2b, from 200 to 1000 drops.

### Charge on Lubricant-Infused Surfaces

**Figure 3:**
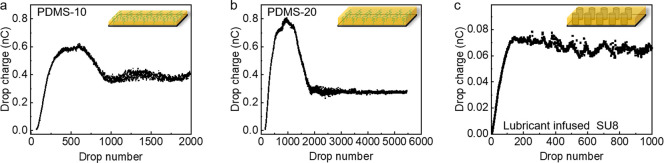
Slide electrification on lubricant-infused surfaces. Drop charge as a function of drop number on (a) PDMS-10, (b) PDMS-20, and (c) lubricant-infused SU8. Inset shows a schematic of (a) PDMS-10, (b) PDMS-20, (c) lubricant-infused SU8. Surface tilted angle = 50°. Water drop volume = 45 μL.

In contrast to PDMS surfaces, on lubricant-infused PDMS the first drops carried no detectable charge (Figures 3a and 3b). Whether no charge separation occurs at all (e.g., due to the different flow profiles near the contact line) or if charges on the lubricant are so mobile that they recombine with charge in the drop is not clear. For low drop numbers (*n* < 100) any charge deposited on the fluid lubricant layer is mobile and could immediately recombine. As a result, water drops do not charge at all. With increasing drop number, the drop charge on lubricant-infused PDMS increased first, showed a maximum and finally decreased to reach a saturation value. In the examples shown in Figures 3a and 3b, the maximum charge was 0.60 nC for PDMS-10 and 0.80 nC on PDMS-20. After 1000 and 2000 drops, it saturated at 0.38 nC ± 0.02 nC and 0.28 nC ± 0.01 nC, respectively. On PDMS-5 and PDMS-15 (Figure S5 in the Supporting Information), they showed a close maximum charge of 0.70 nC ± 0.03 nC, and a similar saturated charge of 0.25 nC ± 0.03 nC. On lubricant-infused SU8, the drop charge also started from ca. 0 nC. Afterward, it underwent increase, decrease, and saturation (Figure 3c). For the SU8 surfaces without lubricant, the drop charge (starting from ca. 0.03 nC) showed the same trend as that on pristine PDMS surfaces (Figure S6 in the Supporting Information).

**Figure 4:**
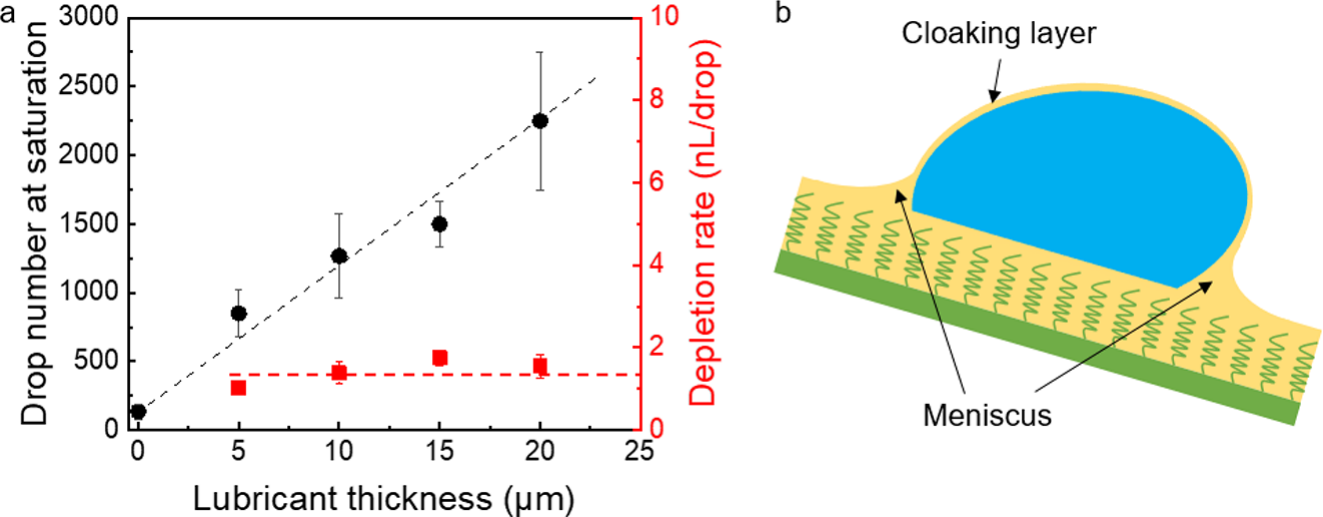
(a) Drop number at saturation and depletion rate on different surfaces. Drop number at saturation means, after that drop number, the drop charge starts to be saturated. (b) Schematic showing the cloaking layer and meniscus.

We explain this dependence of drop charge-versus-drop number by a depletion of lubricant and a resulting change of ion mobility (see below). For this reason, we first analyze the depletion of lubricant. Figure 4 shows the drop number, at which charge saturation is reached, as a function of lubricant thickness. The drop number at saturation increased with the increasing lubricant thickness. This indicates that the charge process is associated with the lubricant depletion on the PDMS surfaces. We further calculated the depletion speed by dividing the depleted lubricant volume by the drop number at saturation. The total volume of depleted lubricant was estimated by *LwD*, where *L* is the slide length, w the width of the drop contact area and *D* the initial film thickness. It shows a constant depletion speed of 1.4 nL/drop ±0.3 nL/drop. As schematically shown in Figure 4b, an annular wetting ridge and a cloaking layer are always formed when water drops slide on the lubricant-infused surfaces.^17,35,36^ Therefore, the lubricant-depletion may be caused by the cloaking layer and meniscus.

To put the 1.4 nL/drop into perspective, we considered the case that the entire lubricant is removed as a cloaking layer, neglecting the meniscus. We assumed that the entire volume of 1.4 nL is contained in a homogeneous layer of thickness *d* on the drop surface. At an apparent contact angle of 98° ± 2° and a drop volume of *V* = 45 μL the free surface area was *A* = 61 mm^2^, leading to *d* = *V*/*A* = 23 nm ± 5 nm. Thus, we estimate that every drop takes a layer of 23 nm away. On the time scale of drops sliding down the sample of ∼0.2 s such a layer can easily form driven by surface tension gradients (Marangoni effect).

We conclude that on an intact lubricant layer no charge is separated. With increasing drop number and decreasing thickness of the lubricant, surface conductivity also decreases. The drops get a chance to keep their drop charge and charging increases. Behind the drops, however, conductivity is still high enough to allow the surface to neutralize before the next drops comes. This fast neutralization of the surfaces allows subsequent drops to acquire a high charge because they are not limited by the surface charges deposited by previous drops. Eventually, for *n* > 1000, surface conductivity is so low that charges deposited by previous drops are still present and limit charging.

Quantitatively, this is described by the model in our previous work.^3^ The charge acquired by a drop in steady state under steady-state conditions is
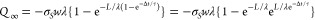
2Here, σ_S_ is the surface charge density deposited by the first drop to the substrate at the beginning of its path, *w* is the width of the contact area of the drop, λ is a characteristic decay length of typically 1 cm, *L* is the slide length of the drop, Δ*t* is the interval between subsequent drops, and τ is the relaxation time for surface charge neutralization. The message of the equation is drop charge is maximal if the surface has a chance to discharge between two subsequent drops. For drop number above *n* = 300–1000 the opposite happens. Surface conductivity decreases, τ increases above Δ*t* = 2 s and the steady-state charge decreases to a value as for the pristine PDMS surface.

In addition, the meniscus of lubricant formed around the drop periphery may change the flow profile of water near the contact line. Since charge separation is attributed to the flow driving away the counterions in the electric double layer, a changed flow profile near the contact line may change charge separation.

### Drop Velocity on Lubricant-Infused Surfaces

**Figure 5:**
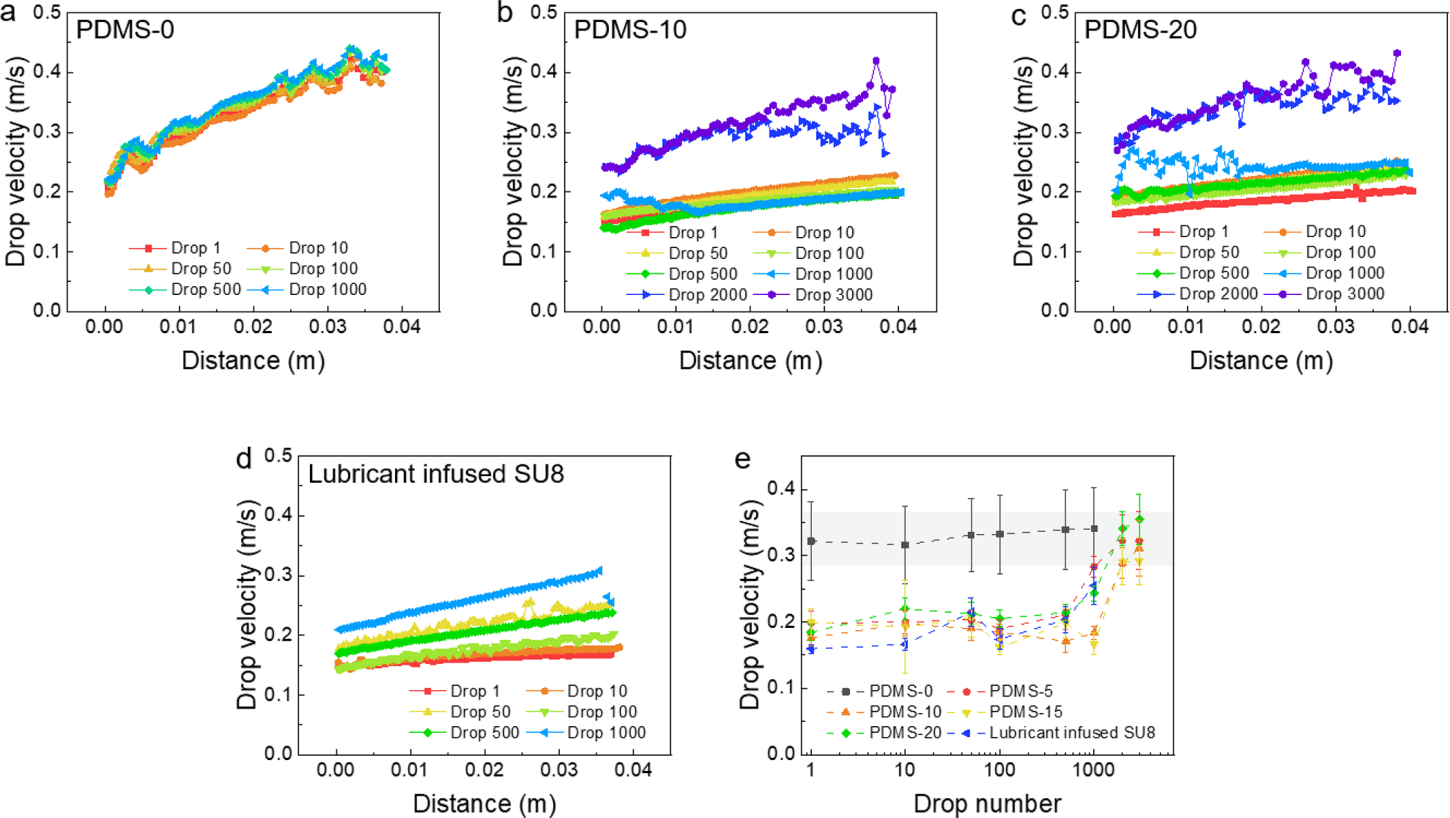
Drop sliding velocity on (a) PDMS-0, (b) PDMS-10, (c) PDMS-20, and (d) lubricant-infused SU8. (e) Concluded average drop velocity as a function of drop number on lubricant-infused surfaces. Surface tilted angle = 50°. Water drop volume = 45 μL.

To further verify the depletion process, we measured the sliding velocity of continuous drops on lubricant-infused surfaces (Figure 5). Similar with the charge results, the velocity of drops on lubricant-infused surfaces was different from that on pristine PDMS. On PDMS-0, the drop reached a velocity of 0.40 m/s ± 0.01 m/s after sliding 4 cm at a tilt angle of 50°. On all lubricant-infused PDMS surfaces, the drop velocity of the first 1000 drops only reached 0.24 m/s ± 0.04 m/s after 4 cm. We attribute the lower velocity of water drops on lubricant-infused surfaces to viscous dissipation caused by meniscus formation and movement of the meniscus.^37,38^ In addition, a Marangoni effect caused by the flow in the sliding drop and a resulting variation in the thickness of the PDMS cloaking layer may lead to an increased friction of drops. Such a Marangoni effect is largely independent of clocking layer thickens, this is constant with our experimental results (Figure S7 in the Supporting Information). Meanwhile, it has been demonstrated before that even tiny gradients in the surface tension of the liquid can induce substantial changes in dynamic contact angles and thus drop motion.^39^ After 1000 drops, the drop velocity started to increase, and finally, it reached a velocity of 0.39 m/s ± 0.03 m/s after 4 cm, which is close to that on PDMS-0. This verifies that the continuous drop sliding on the surface removes lubricant. After enough drops, the lubricant would be consumed, and the surface would turn back to pristine PDMS. For comparison, we also studied lubricant-infused SU8 micropillar arrays (Figure 5d). The velocity of water drops started from 0.16 m/s ± 0.01 m/s for the first drop, and increased to 0.25 m/s ± 0.03 m/s after 1000 drops. This is consistent with that on lubricant-infused PDMS.

## Conclusions

Spontaneous charging of water drops sliding over lubricant-infused surfaces shows a characteristic dependence on drop number. The first drops in a series (*n* < 100) are not or only little charged. We attribute this weak charging to a high mobility of charges on a lubricant layer, which effectively prevents charge separation. The lubricant then is depleted. In parallel, the charge per drop goes through a maximum. This maximum is reached when the mobility of ions is low enough to allow for spontaneous charging but high enough to discharge between subsequent drops. Finally, the charge per drop decreases until it saturates. The saturated charge per drop is similar as that on nonlubricant hydrophobic surfaces. The investigations here not only provide a fundamental understanding of drop charge on lubricant-infused surfaces, it may also depict a guideline (e.g., by adjusting surface conductivity) to optimize the devices for more-efficient electrification.

## Supporting Information Available

Measured drop current curve on PDMS surface; preparation schematic of lubricant infused PDMS; intensity curve of the lubricant; SU8 images; charging curve of water drop on PDMS-5, PDMS-15 and SU8; water drop velocity on surfaces with different lubricant thickness (PDF)
