# Supplementary material for: Spontaneous Charging of Drops on Lubricant-Infused Surfaces
Source: Langmuir. 2022 Oct 3;38(41):12610–6. doi: 10.1021/acs.langmuir.2c02085 (PMC9583601; doi:10.1021/acs.langmuir.2c02085)
Supplement: Supplementary file 1 — la2c02085_si_001.pdf [file la2c02085_si_001.pdf]

Supporting Information

Spontaneous charging of drops on lubricant-infused surfaces

Shuai Li, Pravash Bista, Stefan A. L. Weber, Michael Kappl, Hans-Jürgen Butt*

Max Planck Institute for Polymer Research, Ackermannweg 10, 55128 Mainz, Germany.

*Corresponding author. E-mail: butt@mpip-mainz.mpg.de

This file includes:

Figure S1 to S7

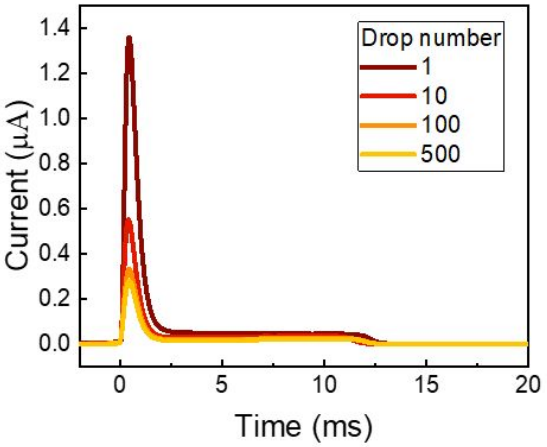

Figure S1. Measured drop current versus time for drop 1, 10, 100, and 500 on PDMS surface.

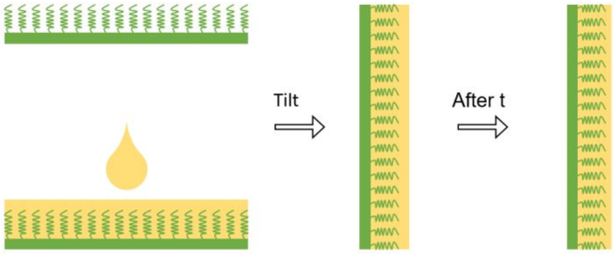

Figure S2. Preparation of lubricant infused PDMS.

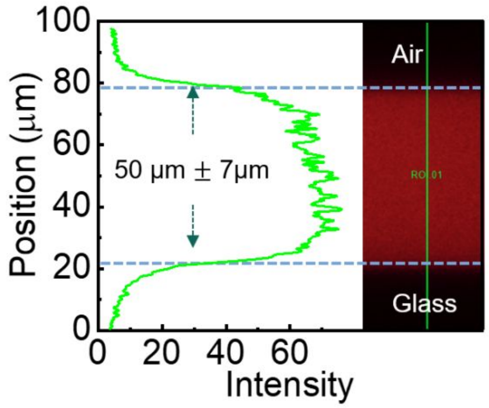

Figure S3. Intensity curve of the lubricant in z direction of surface PDMS-50.

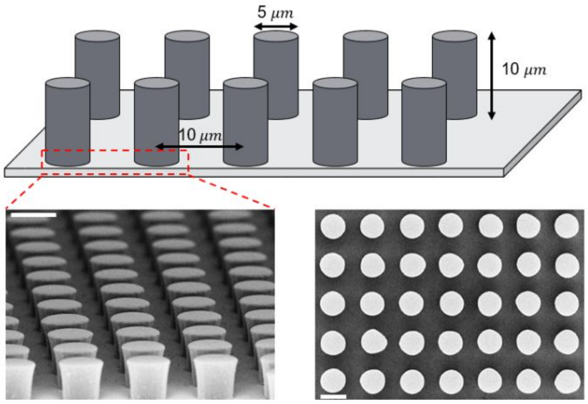

Figure S4. Schematic of SU8 and its SEM images. Scale bar: 10 μm.

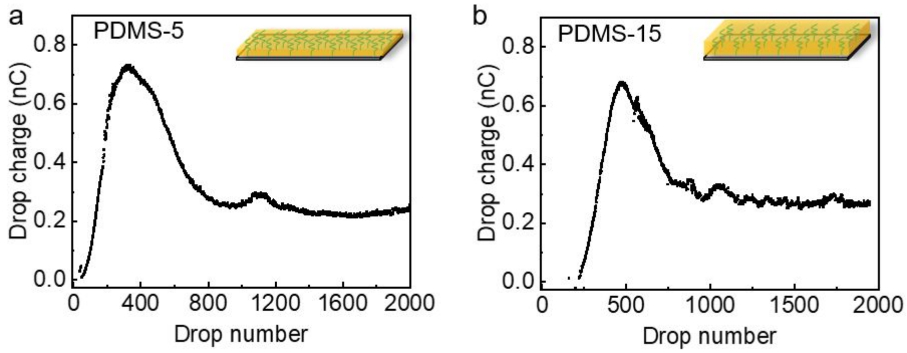

Figure S5. Charge of water drop on PDMS-5 and PDMS-15.

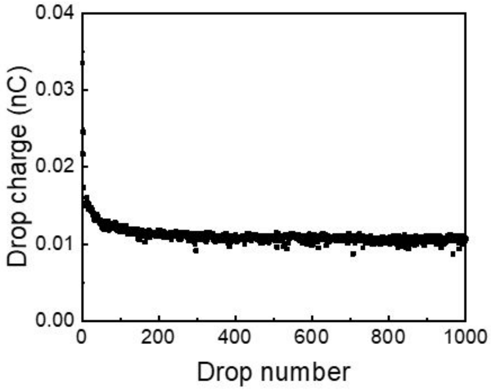

Figure S6. Charge of water drop on SU8 without lubricant.

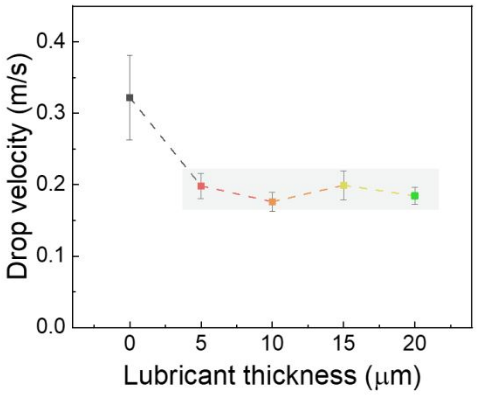

Figure S7. Water drop velocity on surfaces with different lubricant thickness.
